# Regular Exercise and Depressive Symptoms in Korean Older Adults

**DOI:** 10.3390/ijerph18010303

**Published:** 2021-01-03

**Authors:** Young-Mee Kim, Sung-il Cho

**Affiliations:** Department of Public Health Science, Graduate School of Public Health and Institute of Health and Environment, Seoul National University, Seoul 08826, Korea; hihi415@snu.ac.kr

**Keywords:** exercise, depression, hand strength, aged

## Abstract

Prior studies have found that exercise has a positive effect on depressive symptoms in the general population. For older individuals, however, the association between exercise and depressive symptoms is conclusive. We examined whether regular exercise is related to depressive symptoms in 5379 Korean adults aged ≥55 years using data from a 2016 survey administered in the Korean Longitudinal Study of Aging. We used the 10-item Center for Epidemiological Studies–Depression scale to assess depressive symptoms. We performed a multivariate logistic regression analysis to investigate the relationship between regular exercise and depressive symptoms, adjusting for sociodemographic characteristics, self-rated health, number of chronic diseases, body mass index, hand-grip strength, physical disability, cognitive impairment, and health behavior. Interaction terms, including regular exercise and health-related factors, were also added. We found that a lack of regular exercise was significantly related to an increased frequency of depressive symptoms (OR = 1.18, 95% CI = 1.03–1.35). Moreover, hand-grip strength may increase the effect of regular exercise on depressive symptoms in individuals 65 years and older (OR = 1.01 vs. 1.70, 95% CI = 1.05–1.96). Our results suggest that it is important to encourage older individuals to exercise regularly as a means of relieving depressive symptoms.

## 1. Introduction

Worldwide, the segment of the population aged ≥65 years is increasing faster than any other age group. According to data from the 2019 Revision of World Population Prospects [1], persons aged ≥65 years exceeded children under 5 years old in 2018, and by 2050, it is predicted that there will be more than twice as many elderly persons as children under age 5. The fraction of persons aged ≥65 years globally is expected to reach 12% by 2030, 16% by 2050, and 23% by 2100 [1]. In Korea, the population is aging at an unprecedented rate. The fraction of the population aged ≥65 years has risen from 7.3% in 2000 to 14.2% in 2018 [2], and it is projected to grow to 20.3% by 2025 [3]. Moreover, persons aged ≥65 years are expected to compose 43.9% of Korea’s total population by 2060 [3].

Aging is a risk factor for most chronic diseases, decline in physical function, and mental dysfunction [4]. Of persons aged ≥65 years across Organization for Economic Co-operation and Development (OECD) countries, an average of 58% report having two or more chronic diseases [5]. In addition, approximately 15% of the world’s older population suffers from a mental disorder [6], and the number of older adults with mental disorders is projected to double by 2030 [7]. Depression in older adults is the most prevalent and serious mental health problem facing modern society [8]. Approximately 2% of adults aged ≥55 years have major depression, and its prevalence increases with age [9]. Additionally, 10–15% of older adults experience clinically significant depressive symptoms, even in the absence of major depression [10]. Compared to young adults, depressed older adults are at a high risk of suicidal ideation and tend to complete suicide [11]. The suicide rate in Korean older adults is threefold higher than the OECD average, and is the highest of the OECD nations [12].

Risk factors for developing depression have been well established [13]. Sociodemographic factors of age, gender, education level, and income and health-related factors such as self-rated heath, chronic health conditions, and smoking have consistently been identified as important risk factors for depressive symptoms and depression [14,15,16,17]. Previous epidemiological studies on depression have shown that older adults have additional risk factors related to aging such as physical functioning and cognitive dysfunction [18]. According to the behavioral model of late-life depression [19], changes in health, cognitive ability, and physical ability, combined with stressful life events such as bereavement, financial problems, and physical illness or disability of oneself or a family member, may lead to limitations of activities and thereby a lower rate of positive outcomes, increasing the risk of depression.

Previous studies have also identified exercise as a potent lifestyle factor that plays a critical role in preventing depression and significantly reducing depressive symptoms in clinical as well as nonclinical populations [20,21]. Exercise is defined as “a subset of physical activity that is planned, structured, and repetitive”, with the objective of improving or maintaining physical fitness [22]. A recent cross-sectional study (36,595 participants) showed that engaging in exercise at least 1–2 times per week was related to 25% lower odds of depressive symptoms in middle-aged adults (mean age = 41.7 years) [23]. A review of 49 prospective studies (1,837,794 person-years) showed that people with a high degree of physical activity or its subset, exercise, had 17% lower odds of developing depression than those with a low degree [24]. A meta-analysis of 35 randomized controlled trials (1356 participants) also found that exercise was moderately effective at decreasing depressive symptoms in depressed people [25]. A recent meta-analysis of 30 randomized controlled trials (2110 participants) suggested causal relationships between exercise and depressive symptoms in children and adolescents [26].

Despite the wealth of evidence supporting the positive effect of exercise on depressive symptoms in the general population, the association between exercise and depressive symptoms in older adults is inconclusive [27,28,29,30]. A meta-analysis of 30 prospective controlled trials found no statistically significant effect of exercise on depressive symptoms in the elderly [27]. A cluster-randomized controlled trial (891 participants) showed that a moderately intense exercise program failed to reduce depressive symptoms in elderly residents of care homes [28]. A recent cross-sectional study (586 participants) indicated that exercise was not related to depressive symptoms in older adults [29]. Prior studies on the effects of exercise on depressive symptoms among older adults had a number of limitations. First, most were interventional studies that examined only clinically depressed persons, or included both depressed and non-depressed persons but focused on the short-term effects of exercise on depressive symptoms, often using small and highly selective samples that may not have been representative of the overall population [27]. Second, the association between exercise and depressive symptoms among older adults may be moderated by other risk factors, such as physical functioning, or cognitive dysfunction. Therefore, assessing the roles of these factors is necessary to determine the independent influence of exercise. However, few studies have reported such results [24].

Hence, in the current study, using a unique data set from a nationally representative survey conducted in 2016, we investigated the association between regular exercise and depressive symptoms in Korean older adults by investigating three research questions: (1) Does regular exercise affect depressive symptoms in Korean older adults? (2) Are there gender differences in the relationships between regular exercise and depressive symptoms? (3) Do health-related characteristics modify the effect of regular exercise on depressive symptoms? We evaluated the influence of various health-related potential confounders such as physical functioning, physical limitations, and cognitive impairment, that have not been investigated before. In addition, we adopted a large sample that allowed us to examine interactions between key covariates that modify the effects of regular exercise. This yielded much information for population-wide prevention strategies.

## 2. Materials and Methods

### 2.1. Data

We used cross-sectional data from the 2016 sixth-wave survey of the Korean Longitudinal Study of Aging (KLoSA) performed by the Korean Labor Institute. The KLoSA is a nationally representative longitudinal study of adults aged ≥45 years. The KLoSA has been conducted every even-numbered year since 2006 with the primary purpose of generating the data needed to develop and implement socioeconomic policies to address issues related to population aging [31]. Thus, the KLoSA gathered detailed information on demographics, families, employment status, income, socioeconomic factors, health behaviors, medical history, and health and functional status. Participants were selected using a multistage stratified sample of households that represented the entire Korean population. All participants were interviewed using computer-assisted personal interviewing (CAPI) methods. Detailed information on the approaches to survey and sample design can be found at the KLoSA website (https://survey.keis.or.kr/eng/klosa/klosa01.jsp). Recruitment was limited to adults older than 55 years and for whom data on hand-grip strength (HGS) were available. A total of 5379 persons (81.3% of the total KLoSA sample of 6618), comprising 2334 men and 3045 women, was included in the final analysis.

### 2.2. Measures

#### 2.2.1. Regular Exercise

Participants were asked whether they exercised regularly more than once per week, and ”yes” or ”no” answers were recorded.

#### 2.2.2. Depressive Symptoms

Depressive symptoms were assessed using the short-form (10-item) Center for Epidemiologic Studies–Depression (CES-D10) scale, a brief tool to measure depressive symptoms experienced in the past week. Of the ten items, eight were stated negatively and two were stated positively. Item responses were rated on a four-point scale from 0 (occasionally or less than once a day) to 3 (at all time or from five to seven days) with positive items reverse scored. Each item is coded as 1 if it has a score of 1 or higher. Thus, the 10-item scores ranged from 0 to 10. According to the cutoff validated in previous studies [32,33], the 10-item score was recoded as a binary variable as “No depressive symptoms” (0–3) or “Depressive symptoms” (4–10). The scoring validity and reliability of the CES-D10 and the Korean version of the CES-D have been evaluated [32,34].

#### 2.2.3. Key Covariates

##### Sociodemographic Characteristics

Age (years) was recorded using dummy variables representing 55–64, 65–74, or 75 years and over. Marital status was recorded using dummy variables representing separated, divorced, widowed (0), or living with a spouse (1). Educational level was categorized as primary school or less, middle school, high school, or college and above; household income was classified by quartiles. Subjects were classified as living in a rural area (0) or an urban area (1), and working status was classified as not currently working (0) or currently working (1).

##### Health-Related Factors

We also adjusted for health-related factors related to depressive symptoms, such as self-rated health, work limitations due to health, number of chronic diseases, body mass index (BMI), HGS, physical disability, cognitive impairment, smoking, and alcohol consumption. Self-rated health was assessed by the following questions: “How would you rate your general health status?” There were five possible answers, which were dichotomized as good (excellent, good) or fair/poor (fair, poor, very poor). Work limitations due to health status were measured, beginning with a question about whether subjects had difficulty participating in the labor market because of their health status. We divided respondents into two groups depending on whether they answered ‘yes’ (1) or ‘no’ (0). The number of chronic diseases was assessed by a self-reported disease history. Participants could report one or more of eight physician-diagnosed diseases, including cancer, diabetes, hypertension, lung disease, liver disease, cardiac disorders, cerebrovascular disease, psychiatric disease, and arthritis. BMI was evaluated by dividing body weight by height squared (kg/m^2^), and the degree of obesity was evaluated based on BMI. We classified BMI ≥25 kg/m^2^ as obese, 23–24.9 kg/m^2^ as overweight, and <23 kg/m^2^ as normal or low weight according to the World Health Organization classification for Asian and South Asian populations [35].

Physical functioning was measured by HGS, which has been used in observational cohort studies and clinical settings [36,37]. HGS was measured using a hand-held grip dynamometer (model number NO6103; Tanita Corp., Tokyo, Japan) after instructions were given. Weak HGS was defined as HGS <30 and <20 kg in men and women, respectively [38]. The extent of disability was assessed using the Korean versions of the Activities of Daily Living scale (K-ADL). Participants were asked whether assistance is required when performing activities of daily living such as dressing, bathing, eating, continence, washing face/hands, transferring, and toileting. Participants to need help performing one or more of these activities were defined as having difficulty with ADLs. The K-ADL has been validated in the Korean population [39]. To assess cognitive functional impairment, the Korean version of the Mini-Mental State Examination (K-MMSE) was applied. The K-MMSE contains 19 items with the maximum score of 30 points. Subjects who score more than 25 points are classified as “normal”, those who score 21–24 points as having “possible dementia”, and those with fewer than 20 points as having “confirmed dementia” [40,41]. In this study, participants were divided into normal and cognitive impairment (possible dementia or dementia) groups. The scoring validity and reliability of the K-MMSE have been demonstrated [41]. To account for health behavior factors, we also included smoking and alcohol consumption. Subjects were classified as non-smokers or former smokers (0) or current smokers (1). Alcohol consumption was identified as non-drinker (0) or drinker (1).

### 2.3. Statistical Methods

First, we performed a chi-squared test to examine the relationships between independent variables and depressive symptoms. Second, we assessed potential multi-collinearity problems using the GVIF index by R function vif( ) [42]. All variables showed low values of the index below 1.5 adjusting for the number of parameters. Next, we applied a multivariate logistic regression analysis to investigate the relationships between regular exercise and depressive symptoms, adjusting for confounding variables such as age, gender, marital status, education, household income, residential region, working status, self-rated health, work limitations due to health, number of chronic diseases, BMI, HGS, ADL limitations, cognitive impairment, and health behaviors. In addition, we calculated interaction terms between gender and regular exercise in a multiple logistic regression model to assess gender differences in the effect of regular exercise on depressive symptoms. Finally, to determine whether health-related factors altered the effect of regular exercise on depressive symptoms, we added the following interaction terms for regular exercise and health-related factors to the multiple logistic regression: self-rated health, work limitations due to health, number of chronic diseases, BMI, HGS, ADL limitations, and cognitive impairment. In addition, considering that the age of retirement is around 65 years in Korea, we partitioned the sample into those aged 55–64 years and aged 65 years and older and repeated the moderation analysis in each subsample to assess differences in physical conditions between groups. All statistical analyses were conducted using R software ver. 3.3.

## 3. Results

### 3.1. Sociodemographic and Health-Related Characteristics

The sociodemographic and health-related characteristics of the subjects are summarized in Table 1. Subjects with missing values for income or cognitive impairment were defined as the “no-answer group”. The subjects’ ages ranged from 55 to 102 years, with a mean of 69.56 ± 9.05 years. Of the 5379 subjects, 56.6% were women, 77.6% were married, and 40.6% had been educated to primary school level or less. The majority of subjects resided in urban areas (73.5%) and did not participate in the labor market (60.4%). Regarding health status, 70.2% reported their self-rated health as fair or poor, 30.5% experienced work limitations due to their health status, and 67.3% suffered from at least one chronic disease. In terms of BMI, 27.6% were overweight and 27.1% were obese. In terms of HGS, ADL limitations, and cognitive impairment, 43.5% had weak HGS, 1.7% had difficulty in one or more ADLs, and 22.0% exhibited cognitive impairment. Regarding health-related behaviors, 11.0% and 34.7% were current smokers and alcohol users, respectively. Finally, 36.8% of the subjects reported that they exercised regularly once a week.

### 3.2. Prevalence of Depressive Symptoms by Sociodemographic and Health-Related Characteristics

Table 1 shows the prevalence of depressive symptoms according to sociodemographic and health-related characteristics. Of the 5379 participants, 32.8% suffered from depressive symptoms. Using 55–64 years of age as a reference, we found that the prevalence of depressive symptoms increased with age up to 75 years, and women had a higher prevalence of depressive symptoms (36.1%) than men. Subjects who were divorced, separated, or widowed had higher depression scores (48.2%) than their married counterparts. More participants with less than a primary school education reported depressive symptoms (41.3%) compared to those with a high school or college education, and those in the second quartile of household income reported a higher prevalence of depressive symptoms (41.4%) than those in the third or fourth quartile. Subjects who resided in urban areas had a higher prevalence of depressive symptoms (34.2%) than rural residents, and subjects who currently did not work had a higher prevalence of depressive symptoms (38.8%) than those who did.

Regarding health status, subjects whose self-rated health was fair or poor had a higher prevalence of depressive symptoms (38.3%) than those whose self-rated health was good, and subjects who had two or more chronic diseases had a higher prevalence of depressive symptoms (40.5%) than those who had one or no chronic disease. Obese subjects had a higher prevalence of depressive symptoms (34.3%) than those of normal weight or overweight status. Subjects with weak HGS had a higher prevalence of depressive symptoms (43.0%) than those with normal strength, and those with ADL limitations reported a higher prevalence of depressive symptoms (69.9%) than those without such impairment. Subjects with cognitive impairment reported a higher prevalence of depressive symptoms (54.2%) than those without such impairment. Finally, more subjects who did not engage in regular exercise (35.4%) reported having depressive symptoms compared with those who did regular exercise.

### 3.3. Relationship between Regular Exercise and Depressive Symptoms and the Role of Gender

Regular exercise was significantly associated with depressive symptoms (odds ratio [OR] = 1.18, 95% CI = 1.03–1.35), even after controlling for covariates (Table 2). Moreover, there was no gender difference in the effect of regular exercise on depressive symptoms. In the multiple logistic regression model, the interaction between gender and depressive symptoms was not significant (*p =* 0.08). Regarding sociodemographic characteristics, marital status (OR = 0.55, 95% CI = 0.47–0.64), household income (OR = 0.55, 95% CI = 0.44–0.67), residential region (OR = 1.67, 95% CI = 1.43–1.94), and working status (OR = 0.84, 95% CI = 0.72–0.98) were related to depressive symptoms. Regarding health-related characteristics, self-rated health status (OR = 0.58, 95% CI = 0.49–0.68), work limitation (OR = 1.78, 95% CI = 1.55–2.04), HGS (OR = 1.49, 95% CI = 1.31–1.71), ADL limitations (OR = 1.99, 95% CI = 1.23–3.23), and cognitive impairment (OR = 2.29, 95% CI = 1.95–2.67) were related to depressive symptoms.

### 3.4. Moderating Effect of HGS on the Relationship between Regular Exercise and Depressive Symptoms by Age Group

The results show that HGS significantly moderated the effect of regular exercise on depressive symptoms in the 65 years and older group, as shown in Table 3. A weak HGS increased the association between regular exercise and depressive symptoms in the 65 years and older group (OR = 1.01 vs. 1.70, 95% CI = 1.05–1.96). These results show that individuals 65 years and older who do not engage in regular exercise and who have weak HGS may be more vulnerable to depressive symptoms. By contrast, the interactions of regular exercise with self-rated health, work limitations due to health status, number of chronic diseases, BMI, ADL limitations, and cognitive impairment were not statistically significant.

## 4. Discussion

This study investigated whether regular exercise is associated with depressive symptoms using survey data from a nationally representative sample of Korean older adults. We found that regular exercise once a week was significantly negatively related to depressive symptoms in Korean older adults, even after controlling for several sociodemographic and health-related factors that affect depressive symptoms [18]. In addition, there was no gender difference in the regular exercise—depressive symptoms association. Furthermore, our findings show that a weak HGS increased the strength of the association between regular exercise and depressive symptoms, which, to the best of our knowledge, has never been reported previously.

Regarding the mechanism underlying the association, several can be postulated. Depression is related to structural brain abnormalities, including reductions in hippocampus, prefrontal cortex, orbitofrontal, and anterior cingulate cortex volumes and white matter integrity [43]. The hippocampus, which is involved in the processing of depression (such as emotion processing, social cognition, and stress regulation), is the most affected area in depressed individuals [44,45]. Cell biological and biochemical studies have shown that exercise increases the circulation of several neurotrophic factors including brain-derived neurotrophic factor (BDNF), fibroblast growth factors (FGF), insulin growth factor 1 (IGF-1), and vascular endothelial growth factor (VEGF) [46]. By increasing the supply of neurotrophic factors, exercise invigorates a cascade of cellular mechanisms that generate changes in brain structure and function, including in the hippocampus [43]. Exercise also increases the availability of serotonin and norepinephrine, regulates HPA-axis activity, reduces systemic inflammatory signaling, and increases resilience to oxidative stress, all of which may contribute to decreasing depressive symptoms [43,46]. In addition, exercise has several psychosocial benefits that may affect depressive symptoms. Exercise stimulates self-esteem and self-efficacy, which can affect depressive symptoms [47,48]. Moreover, exercise can improve social support by providing opportunities for interaction and socialization [49].

Consistent with previous studies, we found that poor physical functioning, as measured by HGS, ADL limitations, and cognitive impairment were significantly related to depressive symptoms in Korean older adults, even after adjusting for several sociodemographic and health-related covariates. Depressive symptoms are more frequent among the oldest old [50]. This higher frequency is explained by factors associated with aging, such as greater physical disability and increased cognitive impairment [51]. Previous empirical studies have reported that health conditions associated with aging, especially reduced physical functioning [52], disability [53], and cognitive impairment [54], are risk factors for the onset of depressive symptoms and disorders among older adults. These constitute major stressors that lead to a loss of perceived control and lower self-esteem [55]. For example, decreased physical function and the awareness of cognitive impairment increases fear of falling and the psychological reaction to the loss, respectively [56,57]. Decreased physical function is linked to a loss of independent functioning and an increased rate of disability [58]. Disability and cognitive impairment may increase the number of negative life events and reduce the ability to engage in one’s usual social roles and maintain social contacts, which can lead to isolation and a reduced quality of social support, further increasing the risk of depressive symptoms [59,60,61].

Although an understanding of moderators is important because it enables identification of subgroups that could particularly benefit from exercise, there is little empirical evidence for moderators of the association between exercise and depressive symptoms. As mentioned above, most studies of the effects of exercise on depressive symptoms have been limited by a small sample size [27]. In contrast, we employed a nationally representative survey dataset that contains various subpopulations, which allowed us to investigate how the impact of exercise on depressive symptoms varies with sociodemographic and health-related factors. In this study, HGS moderated the association between regular exercise and depressive symptoms in the 65 years and older group, indicating that the effect of regular exercise on depressive symptoms was stronger in participants with a weak HGS relative to those with a normal HGS after controlling for sociodemographic characteristics and health-related covariates. HGS is reported to reliably represent overall muscle strength and comprehensive physical performance in older adults [36]. Our data support the claim that the benefit of exercise is greatest in individuals with greater loss of physical function [51,62]. Health professionals needs to have a well-rounded understanding of the barriers to and enablers of exercise for older adults with greater loss of physical function, including a weak HGS, and develop an exercise plan tailored to the individual’s health status [63].

Gender is another important factor, and most studies have found that women have a higher risk of depressive symptoms than men [64]. In addition, men report more frequent exercise than women [65]. Despite these significant gender differences in depressive symptoms and exercise behaviors, research has yet to settle whether exercise similarly improves men’s and women’s depressive symptoms. Some studies have shown that the effect of exercise on depressive symptoms may varies as a function of gender [25,66], whereas others have found no evidence of gender differences in the relationship between exercise and depressive symptoms [24]. Using data from a nationally representative sample of older adults, we found no gender difference in the effect of regular exercise on depressive symptoms, indicating that the effect of regular exercise may be independent of gender in older people (*p* = 0.08).

Several limitations of our study should be considered. First, we employed a cross-sectional design. Thus, we were unable to elucidate causal link between regular exercise and depressive symptoms in older adults. However, several prospective cohort studies have demonstrated that engaging in exercise was significantly associated with decreased in developing future depression [24]. Second, we used self-reported questionnaires to measure regular exercise, depressive symptoms, and covariates, all of which may be affected by older adults’ memories and mood. Third, the missing indicator method was adopted to address missing data [67]. In our data set, 7.7% of income data was missing and 2.0% of data on cognitive impairment was missing, as identified by the number of “no answer” responses. The results for the logistic regression excluding subjects with missing data were comparable to those for the full analysis (*n* = 4863). Future studies should try to overcome some of the limitations of this study.

## 5. Conclusions

Our findings suggest that regular exercise once a week was associated with a reduced likelihood of depressive symptoms among Korean adults aged 55 years and older after adjusting for sociodemographic characteristics and health-related covariates. Moreover, exercise had a greater effect on depressive symptoms in individuals 65 years and older with a weaker HGS. The findings suggest that healthcare professionals should promote participation in regular exercise among older adults to prevent depressive symptoms, particularly for individuals with a greater loss of physical function. Furthermore, governments and policy makers should attempt to encourage lifelong participation in exercise. Further research is needed, particularly on strategies to promote exercise in older people with greater loss of physical function.

## Figures and Tables

**Table 1:** Sociodemographic and health-related characteristics of the study population and prevalence of depressive symptoms by sociodemographic and health-related characteristics, *n* (%).

	ALL (*n* = 5379)	No-Depressive Symptoms (*n* = 3617, 67.2%)	Depressive Symptoms (*n* = 1762, 32.8%)	*p*-Value ^a^
**Sociodemographic Characteristics**				
Age (years)				
Mean	69.56 ± 9.05			
55–64	1872 (34.8)	1383 (73.9)	489 (26.1)	<0.001
65–74	1818 (33.8)	1252 (68.9)	566 (31.1)	
75 years and older	1689 (31.4)	982 (58.1)	707 (41.9)	
Gender				
Male	2334 (43.4)	1672 (71.6)	662 (28.4)	<0.001
Female	3045 (56.6)	1945 (63.9)	1100 (36.1)	
Marital status				
Separated, divorced, or widowed	1205 (22.4)	624 (51.8)	581 (48.2)	<0.001
Married	4174 (77.6)	2993 (71.7)	1.181 (28.3)	
Education level				
Primary school or less	2185 (40.6)	1282 (58.7)	903 (41.3)	<0.001
Middle school	955 (17.8)	689 (72.1)	266 (27.9)	
High school	1723 (32.0)	1262 (73.2)	461 (26.8)	
College or above	516 (9.6)	384 (74.4)	132 (25.6)	
Household income (quartile)				
1Q (lowest)	1240 (23.1)	774 (62.4)	466 (37.6)	<0.001
2Q	1242 (23.1)	728 (58.6)	514 (41.4)	
3Q	1241 (23.1)	828 (66.7)	413 (33.3)	
4Q (highest)	1241 (23.1)	1013 (81.6)	228 (18.4)	
No answer	415 (7.7)	274 (66.0)	141 (34.0)	
Residential region				
Rural	1423 (26.5)	1012 (71.1)	411 (28.9)	<0.001
Urban	3956 (73.5)	2605 (65.8)	1351 (34.2)	
Working status				
Not currently working	3248 (60.4)	1988 (61.2)	1260 (38.8)	<0.001
Currently working	2131 (39.6)	1629 (76.4)	502 (23.6)	
**Heath status characteristics**				
Self-rated health				
Fair or poor	3776 (70.2)	2331 (61.7)	1445 (38.3)	<0.001
Good	1603 (29.8)	1286 (80.2)	317 (19.8)	
Work limitations due to health status				
No	3736 (69.5)	2765 (74.0)	971 (26.0)	< 0.001
Yes	1643 (30.5)	852 (51.9)	791 (48.1)	
Number of chronic diseases				
No disease	1758 (32.7)	1314 (74.7)	444 (25.3)	<0.001
One disease	1675 (31.1)	1145 (68.4)	530 (31.6)	
Two diseases or more	1946 (36.2)	1158 (59.5)	788 (40.5)	
BMI (kg/m^2^)				
<23	2437 (45.3)	1615 (66.3)	822 (33.7)	<0.001
23–24.9	1483 (27.6)	1044 (70.4)	439 (29.6)	
≥25	1459 (27.1)	958 (65.7)	501 (34.3)	
Hand-grip strength				
Normal	3037 (56.5)	2283 (75.2)	754 (24.8)	<0.001
Weak	2342 (43.5)	1334 (57.0)	1008 (43.0)	
ADL limitations				
No	5286 (98.3)	3589 (67.9)	1697 (32.1)	<0.001
Yes	93 (1.7)	28 (30.1)	65 (69.9)	
Cognitive impairment				
Normal	4090 (76.0)	3028 (74.0)	1062 (26.0)	<0.001
Cognitive impairment	1181 (22.0)	541 (45.8)	640 (54.2)	
No answer	108 (2.0)	48 (44.4)	60 (55.6)	
**Health behaviors characteristics**				
Smoking				
Non or former smokers	4785 (89.0)	3189 (66.6)	1596 (33.4)	<0.001
Current smokers	594 (11.0)	428 (72.1)	166 (27.9)	
Alcohol consumption				
Non-drinker	3513 (65.3)	2269 (64.6)	1244 (35.4)	<0.001
Drinker	1866 (34.7)	1348 (72.2)	518 (27.8)	
Regular exercise				
No	3401 (63.2)	2197 (64.6)	1204 (35.4)	<0.001
Yes	1978 (36.8)	1420 (71.8)	558 (28.2)	

Note: Percentages in the shaded areas are the column percentages of each variable. ^a^ Chi-squared test.

**Table 2:** Associations of regular exercise with depressive symptoms.

	Odds Ratio (95% Confidence Interval)
	Depressive Symptoms
**Sociodemographic characteristics**	
Marital status	
Separated, divorced, or widowed	1.00
Married	0.55 (0.47–0.64) ^‡^
Education level	
Primary school or less	1.00
Middle school	0.86 (0.71–1.04)
High school	0.96 (0.80–1.14)
College or above	1.07 (0.82–1.39)
Income (quartile)	
1Q (lowest)	1.00
2Q	0.95 (0.79–1.14)
3Q	0.85 (0.70–1.02)
4Q (highest)	0.55 (0.44–0.67) ^‡^
No answer	1.12 (0.87–1.44)
Residential region	
Rural	1.00
Urban	1.67 (1.43–1.94) ^‡^
Working status	
Not currently working	1.00
Currently working	0.84 (0.72–0.98) ^†^
**Health status characteristics**	
Self-rated health	
Fair or poor	1.00
Good	0.58 (0.49–0.68) ^‡^
Work limitations due to health status	
No	1.00
Yes	1.78 (1.55–2.04) ^‡^
Number of chronic diseases	
No disease	1.00
One disease	1.06 (0.90–1.25)
Two diseases or more	1.11 (0.94–1.32)
BMI (kg/m^2^)	
<23	1.00
23–24.9	0.95 (0.81–1.11)
≥25	1.01 (0.86–1.17)
Hand-grip strength	
Normal	1.00
Weak	1.49 (1.31–1.71) ^‡^
ADL limitations	
No	1.00
Yes	1.99 (1.23–3.23) ^‡^
Cognitive impairment	
Normal	1.00
Cognitive impairment	2.29 (1.95–2.67) ^‡^
No answer	2.67 (2.08–2.85) ^‡^
**Health behavior characteristics**	
Smoking	
Non or former smokers	1.00
Current smokers	0.89 (0.71–1.12)
Alcohol consumption	
Non-drinker	1.00
Drinker	1.05 (0.90–1.22)
Regular exercise	
Yes	1.00
No	1.18 (1.03–1.35) ^†^

^†^*p* < 0.05, ^‡^ *p* < 0.01. Models were adjusted for all other variables in Table 1.

**Table 3:** Odds ratios for the associations of depressive symptoms and regular exercise with HGS including significant interaction effects.

		Odds Ratio (95% Confidence Interval)	
		Regular Exercise	
		Yes	No
Age	Hand-grip strength		
55–64	Normal	1	1.10 (0.82–1.43)
	Weak	1.32 (0.86–2.02)	1.79 (0.78–2.25)
65 years and older	Normal	1	1.01 (0.75–1.23)
	Weak	1.17 (0.87–1.44)	1.70 (1.05–1.96) ^†^

^†^*p* < 0.05. Models were adjusted for all other variables in Table 1.

## Data Availability

Publicly available datasets were analyzed in this study. This data can be found here: https://survey.keis.or.kr/klosa/klosa01.jsp.
